# Recurrent Wheezing and Cough Caused by Double Aortic Arch, Not Asthma

**DOI:** 10.1155/2017/8079851

**Published:** 2017-07-25

**Authors:** Qiao Zhang, Zhou Fu, Jihong Dai, Gang Geng, Wenlong Fu, Daiyin Tian

**Affiliations:** Department of Respiratory Medicine, Children's Hospital of Chongqing Medical University, Ministry of Education Key Laboratory of Child Development and Disorders, Chongqing 400014, China

## Abstract

**Introduction:**

Double aortic arch is a congenital vascular abnormality in which the connected segments and their branches course between and compress the trachea and esophagus, often resulting in invariable airway compression.

**Case Presentation:**

A 4-year-old boy with a history of recurrent wheezing was admitted to our hospital for evaluation of asthma based on his past medical history, persistent cough, wheezing, and airway hyperresponsiveness by lung function test. Double aortic arch was diagnosed with computed tomography angiogram. After surgery, the respiratory infection improved strikingly. Early diagnosis and treatment may prevent chronic, irreversible complications.

**Conclusion:**

We present a case of double aortic arch masquerading as asthma.

## 1. Introduction

Congenital abnormalities of the aortic arch and its major branches result in the formation of vascular rings around the trachea and esophagus with varying degrees of compression [1]. One of the most common anomalies is double aortic arch. In this type of vascular ring, the ascending aorta bifurcates and forms a right and left sided arch [2]. As the most common congenital anomaly of the aortic arch system, the connected segments and their branches course between and compress the trachea and esophagus [3–7]. Symptoms are frequently present during childhood. Affected children may have cough, stridor, dyspnea, and upper respiratory tract infections. It can be diagnosed with the aid of echocardiography, axial computed tomography, and magnetic resonance imaging [8–12]. Bronchoscopy may be helpful in more severe cases to determine the extent of airway narrowing. As for the treatment, surgery is advised for symptomatic patients who have evidence of tracheal compression [1].

Asthma is a chronic inflammatory condition of the airways resulting in episodic airflow obstruction. Intermittent dry coughing and expiratory wheezing are the most common chronic symptoms of asthma. The diagnosis of asthma in children requires a careful review of their past medical history, family history, a physical examination, and lung function test [13].

The diagnosis of double aortic arch is typically made because of the symptoms caused by tracheal and/or esophageal compression. Children always present with cough, biphasic stridor, wheezing, recurrent respiratory infections, and dysphagia [14]. The diagnosis of asthma is often based largely on clinical symptoms: hyperresponsiveness and reversibility of airflow obstruction. Therefore, the diagnosis of asthma is very often based on a clinical history and a patient's response to a medication [15], that is, the point in the differential diagnosis between these two diseases.

This is a case report of a patient with double aortic arch that had been misdiagnosed as asthma.

## 2. Case Presentation

A 4-year-old boy with a history of recurrent wheezing was admitted to the inpatient department of our hospital for evaluation of acute exacerbation of severe asthma. On admission, the patient complained of recurrent wheezing and intermittent cough, with shortness of breath, general fatigue, orthopnea, being pale, and sweating. All his symptoms can worsen at night. The child's parents denied any foreign body aspiration, weight loss, and trauma. Physical examination revealed tachypnea, cyanosis, suprasternal retraction, and a prolonged expiratory crackles at lungs. His saturation level of oxygen in hemoglobin was 91% with an oxygen supplemental therapy. In laboratory tests, both his skin prick test and serum dosage of IgE were negative, and his lung function test showed airway hyperresponsiveness, in which his V75 and V50 were in mild reduction, PF was in moderate reduction, and the bronchial dilation was negative.

The child has a history of recurrent respiratory infection (4 times), which is characterized by cough, fever, and wheeze. One was at the age of 10 months and three were all after 3 years. He was diagnosed as asthma because of a history of recurrent wheezing episodes and airway hyperresponsiveness suggested by lung function test. But his skin prick test and serum dosage of IgE did not support this diagnosis. However, the patient did not do regular follow-up. During the age of 10 months to 3 years, there was no wheezing. His wheezing could partially be improved by bronchodilator and systemic corticosteroid. Each episode of wheezing had lasted more than 20 days when the patient was treated in outpatient department. It seemed that the patient is not so sensitive to the steroid treatment.

Considering the patient's history, this was his fourth asthmatic attack, but this time when he was referred to the inpatient department, he was not relieved of the symptoms by routine treatment. Should we need to consider other diseases rather than asthma? After admission, we did the bronchoscopy to examine his airway. The bronchoscopy revealed tracheal narrowing at the lower end of the trachea without any internal pathology. The normal CT scan of lung showed no parenchymal disease. Then we advised the patient to do an enhanced computed tomography angiogram to get more information about the airway and the vessel. Considering some other social factors and radiation, the parents refused to do so. After 8 days of treatment the patient's parents asked for discharging from the hospital.

**Figure 1:**
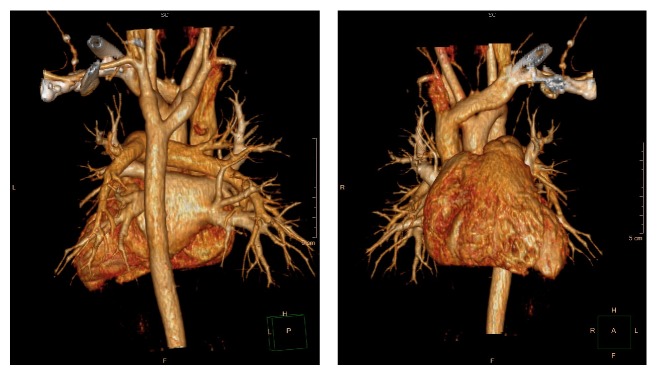
Revascularization on the heart showing the double aortic arch of this case; the right aortic arch passed by the posterior of trachea and esophagus and then joined the descending aorta.

**Figure 2:**
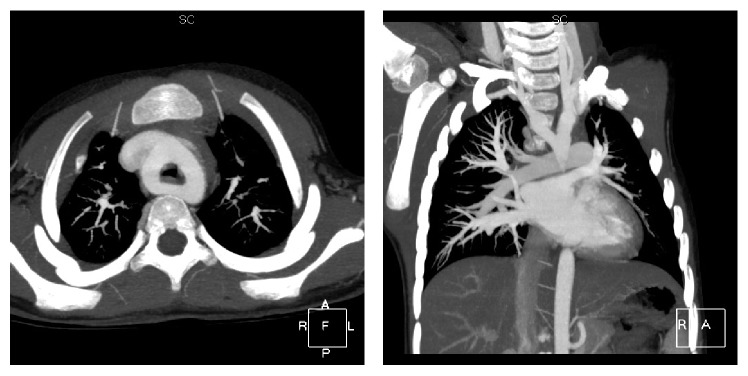
Contrast-enhanced CT showing the double aortic arch.

When we did follow-up two weeks later, the patient's parents finally agreed to do an enhanced computed tomography angiogram on the lung. The diagnosis of DAA was confirmed. CT angiogram revealed a vascular ring, consisting of a double aortic arch, around the trachea, and the right side was dominant which was compressing the trachea. The medical images of this patient are shown in Figures 1 and 2. The echocardiogram confirmed the presence of the vascular ring. The inner diameter of right and left aortic arch is 9.3 and 9.9 mm. The double aortic arch gives off the common carotid artery and subclavian artery to the left descending aorta.

Three months later, the child was scheduled for surgery to correct the vascular ring. After oxygenation with 100% oxygen, anesthesia was induced, the triangle of ductus arteriosus was exposed by pulling the left lung, then an incision was made on the mediastinal pleura at the surface of descending aorta to make the left and right aortic arch free, and finally the right aortic arch was divided at the joint to relieve the compression to trachea and esophagus. The child was ventilated mechanically for 12 hours in Intensive Care Unit. He was relieved of symptoms without any stridor. Then we reperformed a CT scan and airway remodeling on the chest, which showed a slighter narrowing in the lower trachea. But the child refused to do a follow-up check on his lung function test. Then he was discharged on his seventh postoperative day. At follow-up evaluation performed six months after surgery, the patient was free of respiratory problems and pulmonary infections.

## 3. Discussion

In this case, a four-year-old boy with a history of recurrent wheezing and coughing is presented. The patient was initially misdiagnosed as severe asthma attack based on his past medical history, persistent symptoms of cough, recurrent wheezing, and airway hyperresponsiveness suggested by lung function test. While the computed tomography angiogram confirmed double aortic arch, the child had tracheal compression due to the DAA leading to the recurrent wheezing and repeated respiratory infection.

Double aortic arch is a rare congenital vascular malformation. DAA may be of right dominant (70% case), left dominant (25% case), and balanced type (5%). The ring of the DAA causes the compression of the airway and induced recurrent wheezing [16]. These rare congenital vascular disorders cause a complete or partial encirclement of trachea and esophagus. An early monograph from the Mayo Clinic divided vascular rings into 7 types, but over 95% of cases can be classified in 4 main categories: double aortic arch, right arch/left ligament, innominate artery compression, and pulmonary artery sling. DAA is the most common form of complete vascular ring, which originates when the ascending aorta bifurcates into a right and a left arch, both usually patent; these arches encompass trachea and esophagus and rejoin in the thoracic descending aorta. Symptoms such as stridor, persistent cough, recurrent wheezing, and respiratory infections usually lead to diagnosis and surgical treatment in childhood [17–20].

Regarding this case, the boy had recurrent wheezing four times, intermittent cough, shortness of breath, orthopnea, paleness, tachypnea, cyanosis, and a prolonged expiratory crackles at lungs. Those symptoms were more often after age 3, and his lung function test showed airway hyperresponsiveness. Bronchodilator and systemic corticosteroids administration seemed to be partially beneficial. All those were why he was misdiagnosed as asthma for a long time. But it seemed he had something more we could not explain as asthma. His skin prick test and serum dosage of IgE were both negative. His response to the corticosteroid was not as sensitive as other asthma patients. So if an asthma patient was not relieved of symptoms with routine treatment, differential diagnosis is necessary to figure out if other reasons are hiding.

## 4. Conclusion

Double aortic arch is a rare congenital vascular malformation, but it still causes many misdiagnoses of recurrent wheezing as asthma. The case emphasizes the suspicion for vascular ring due to DAA in children with repeated wheezing and cough when other common etiologies were excluded. Detailed history-taking, following up the treatment, and differential diagnosis are very important for final correct diagnosis.
